# P-768. Clinical Complications Among Patients with Complicated Urinary Tract Infections Treated with Intravenous Carbapenems in Hospital: a US Cohort Study

**DOI:** 10.1093/ofid/ofaf695.979

**Published:** 2026-01-11

**Authors:** Ryan K Shields, Myriam Drysdale, Rose Chang, Louise Yu, Maral DerSarkissian, Megan Pinaire, Zhuo Chen, Mei Sheng Duh, Alanna Farrell-Foster, Fanny S Mitrani-Gold

**Affiliations:** University of Pittsburgh, Pittsburgh, PA; GSK, Brentford, Middlesex, England, United Kingdom; Analysis Group, Inc., Boston, Massachusetts; Analysis Group Inc., Boston, MA, United States, Boston, Massachusetts; Analysis Group, Inc., Boston, Massachusetts; Analysis Group, Inc., Boston, Massachusetts; Analysis Group, Inc., Boston, Massachusetts; Analysis Group Inc., Boston, MA, United States, Boston, Massachusetts; GlaxoSmithKline plc., Deerfield , IL

## Abstract

**Background:**

Patients with complicated urinary tract infections (cUTIs) caused by multidrug-resistant (MDR) uropathogens often require hospitalization and intravenous (IV) antibiotic treatment. This real-world cohort study aimed to describe clinical complications in patients with cUTI.

**Figure:**
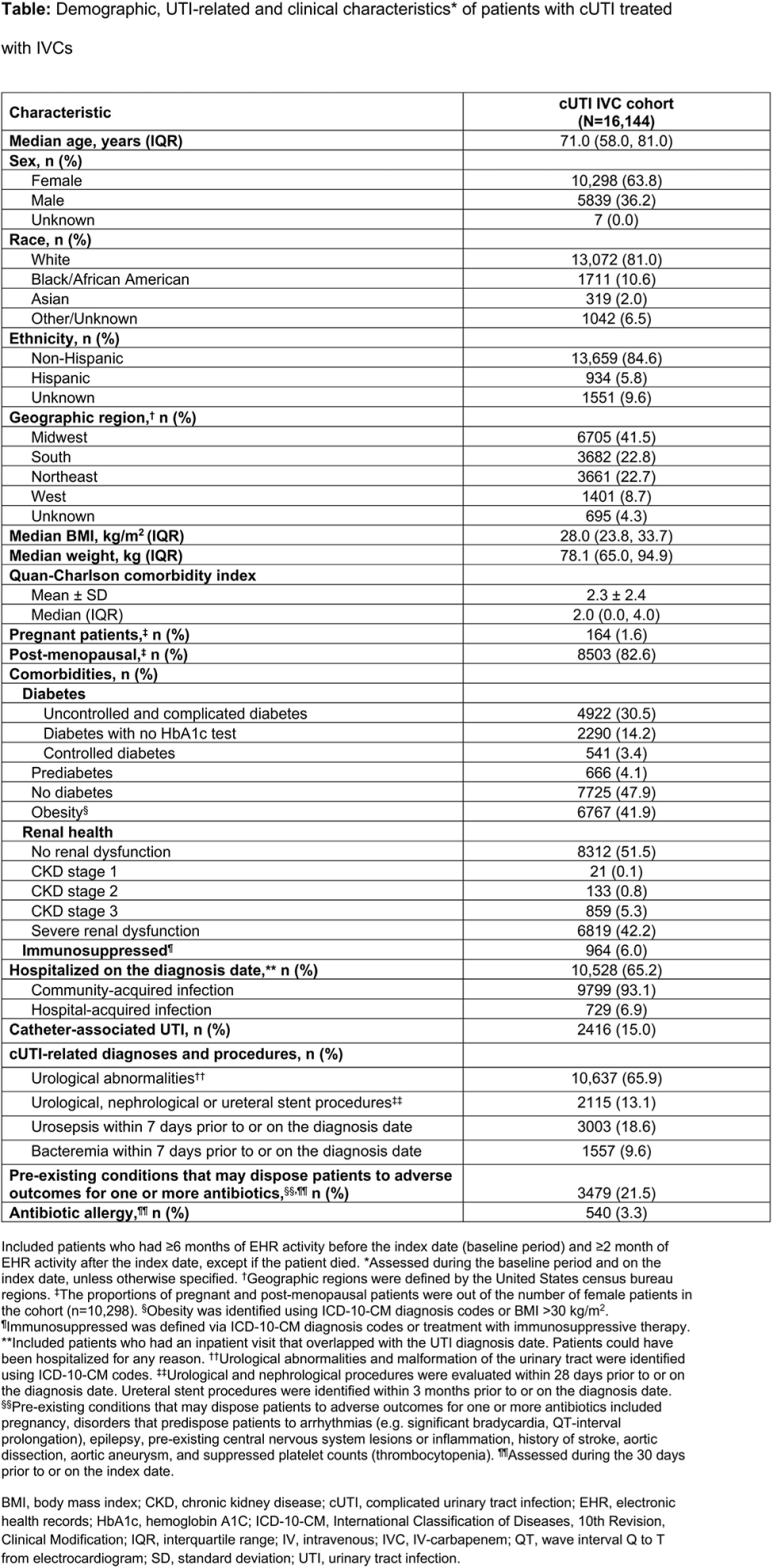

**Figure:**
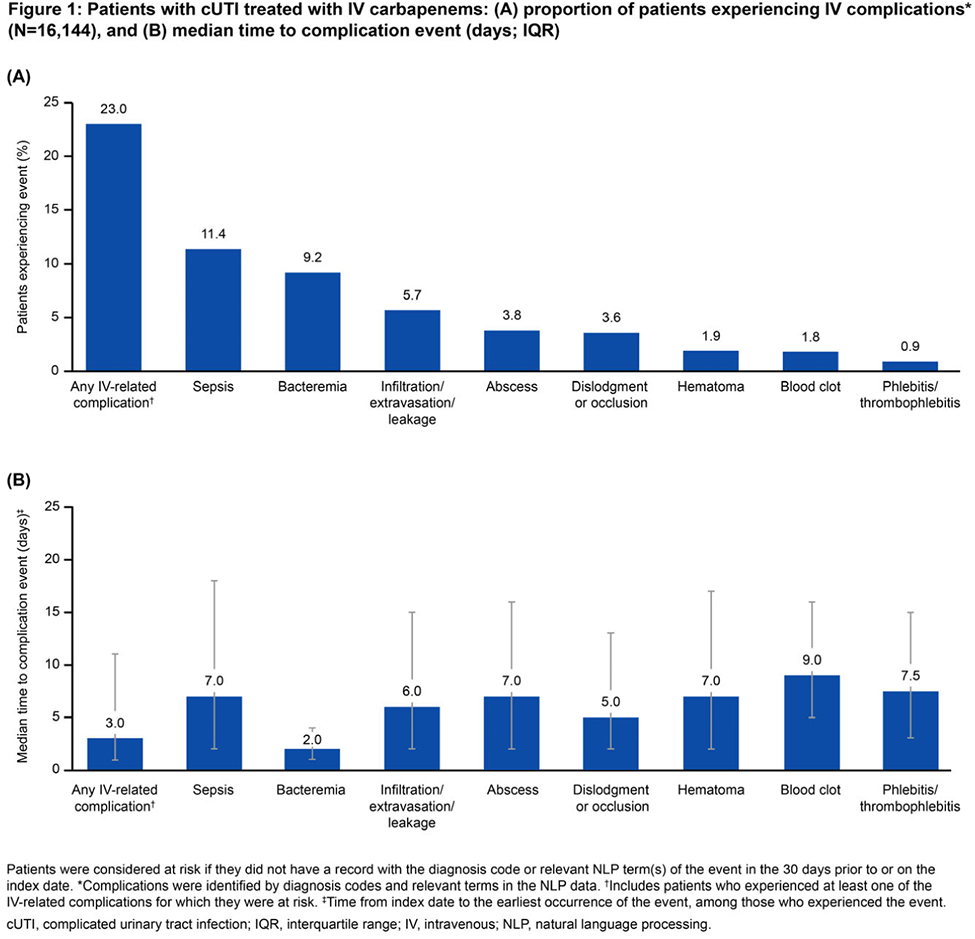

**Methods:**

Adults (≥ 18 years) who received IV-carbapenem (IVC) treatment within 7 days of a primary or admitting UTI diagnosis between January 1, 2018 and September 30, 2023 were identified using Optum’s de-identified Electronic Health Record dataset. These patients had cUTI infection (pyelonephritis, complicated cystitis or urosepsis), with the index date defined as the date of IVC treatment initiation. IV complications were evaluated from index to the earlier of 30 days following index or death. Complications related to extended length of stay (LOS) were evaluated from hospital admission to the earlier of discharge or death among a subgroup of hospitalized patients admitted for cUTI. Clinical complications were assessed overall and by LOS (1–3 vs 4+ days).

**Figure:**
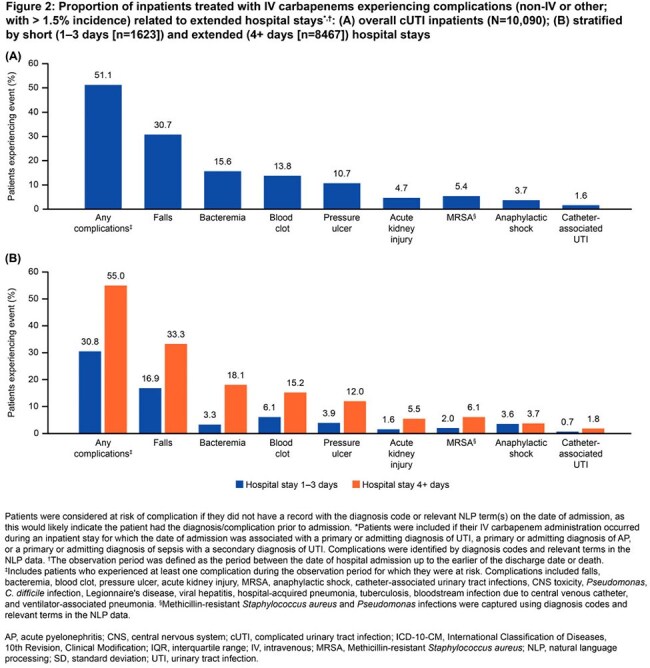

**Results:**

In total, 16,144 patients treated with IVC for UTI were included: median age 71.0 years, 63.8% female, 41.9% obese, 30.5% uncontrolled and complicated diabetes, 42.2% severe renal dysfunction, and median (interquartile range [IQR]) Quan-Charlson comorbidity index of 2.0 (0.0, 4.0; Table). The incidence of any IV-related complication was 23.0% (n=3708), with a median (IQR) time to event of 3.0 days (1.0, 11.0; Figure 1). Among the subgroup of hospitalized patients (n=10,090), median (IQR) LOS was 6.0 days (4.0, 8.0). The incidence of any non-IV-related complications related to hospital LOS was 51.1% (n=5153); the incidence of any complication among patients with a LOS of 1–3 and 4+ days was 30.8% and 55.0%, respectively. The most common complication related to extended LOS was falls (overall, 30.7%; 1–3 days, 16.9%; 4+ days, 33.3%; Figure 2).

**Conclusion:**

Over half of cUTI patients treated with IV carbapenems experienced at least one complication, with higher incidence among patients with extended LOS. These findings highlight the clinical burden of patients treated with IVC and the potential value of new oral treatment options in cUTI.

Funding: GSK study 221138.

**Disclosures:**

Myriam Drysdale, PhD, GSK: Employee|GSK: Stocks/Bonds (Public Company) Rose Chang, ScD, Analysis Group: Employee|GSK: Grant/Research Support Louise Yu, MS, Analysis Group: Employee|GSK: Grant/Research Support Maral DerSarkissian, PhD, Analysis Group: Employee|GSK: Grant/Research Support Megan Pinaire, MPH, Analysis Group: Employee|GSK: Grant/Research Support Zhuo Chen, MPH, Analysis Group: Employee|GSK: Grant/Research Support Mei Sheng Duh, MPH, ScD, Analysis Group: Employee|GSK: Grant/Research Support Alanna Farrell-Foster, GSK: Employee|GSK: Stocks/Bonds (Public Company) Fanny S. Mitrani-Gold, MPH, GSK: Employee|GSK: Stocks/Bonds (Public Company)

